# Using a tablet-based composition marking recording system to conduct think-aloud for composition rating research

**DOI:** 10.1186/s41039-015-0020-2

**Published:** 2015-11-17

**Authors:** Kat Leung, Barley Mak, Howard Leung

**Affiliations:** 1 Caritas Institute of Higher Education, Hong Kong, SAR China; 2 The Chinese University of Hong Kong , Hong Kong, People’s Republic of China; 3 City University of Hong Kong , Kowloon Tong, Hong Kong SAR People’s Republic of China

**Keywords:** Chinese composition rating, Think-aloud, Tablet, Teacher feedback

## Abstract

Language teacher’s composition rating process has become an issue of growing concern in writing research over the past three decades. It is evident that a substantial number of composition rating research have employed concurrent think-aloud protocol analysis (TAPA) as a major method of qualitative data collection and data analysis. For the previous studies that adopted the method of TAPA in exploring the composition marking process, they were mostly confined to the study on L1/L2 English language education and not much has been done specifically on L1 Chinese language education. None of the previous studies employing TAPA were conducted with a computer marking platform. In the past, researchers generally videotaped and recorded teacher participants’ think-aloud protocol during the composition marking process. However, the presence of the camera put a certain amount of psychological pressure on the participant and affected his/her performance in the rating task. To address these research gaps, a camera-free, tablet-based composition marking system is developed allowing writing researchers to examine the composition marking behavior of Chinese writing teachers with the method of TAPA conducted in a tablet-assisted marking environment. The system is able to eliminate the psychological pressure on the teacher participants as in video recording and to record their process of marking compositions systematically for later analysis.

## Introduction

Various new technologies have been proposed to help researchers examine different aspects of teaching the Chinese language. Novel technology has been developed for mobile device to help teachers implement reading strategy instruction and support students’ individual and co-operative reading activities in Chinese language classes (Chang et al. 2010). A measure has been proposed to assess the ability of Chinese character recognition made by foreigner Chinese learners, and a two-phase learning strategy has been presented for these learners to learn Chinese characters (Ho and Lin 2010). A mobile-assisted game was adopted to let students learn the formation of Chinese characters, and the social interactions have been examined to analyze how student grouping can affect the learning outcome (Wong et al. 2013). This paper is focused on the study of the Chinese composition rating process by combining a tablet-based marking recording system with a think-aloud approach.

The composition rating process of language teachers has received a considerable amount of attention in the writing research over the past three decades. However, it is not easy to identify the procedures and mechanism of this process and distinguish its sub-processes. The methodology frequently adopted by writing researchers is think-aloud protocol analysis (TAPA), in which participating teachers are asked to verbalize their thoughts while they are assessing students’ writings. TAPA, if employed appropriately, is generally considered as an effective tool for data collection and data analysis. TAPA has been widely used in many studies on composition rating to examine rater’s decision-making behavior and their rating processes (e.g. Vaughan 1990; Cumming 1990, 2001, 2002; Huot 1993; Dalaruelle 1997; DeRemer 1998; Barkaoui 2010, 2011). Attempts have been made to develop models of composition rating in the contexts of English as a first language (L1) and English as a secondary language (ESL) on the base of data collected from TAPA (Freedman and Calfee 1983; Wolfe 1997, 2006; Lumley 2006). A number of studies have explored and compared rating processes between expert and novice writing teachers (Cumming 1990; Huot 1993; Milanovic et al. 1996; Barkaoui 2011), between L1 English and ESL/EFL instructor (Cumming et al. 2002; Erdosy 2004), or between trained and untrained raters (Weigle 1998, Weigle 1999; Wolfe 1997, 2006). Though the findings reported in these TAPA studies were not consistent, they all employed the method of think-aloud as an instrument for data collection and also included the component of think-aloud protocols in their data analysis.

It can be observed from the existing literature that the extant composition rating research using TAPA was mostly limited to the L1/L2 English language education, while not much has been done specifically on L1 Chinese language education. It is also worth mentioning that teacher participants in the previous think-aloud studies were asked to rate students’ written composition while concurrently doing think-aloud in a paper-based writing assessment environment. To the best of our knowledge, none of the previous researches employing TAPA were conducted with a computer marking platform.

Along with the growing use of on-screen rating of student writings in the high-stake writing assessment both internationally and locally, as suggested by Crisp (2008) and Barkaoui (2010, 2011), we are convinced that investigation in composition raters’ decision-making process in a computer-assisted environment would definitely contribute to the training of public examination rater or even writing teachers in general, and the improvement of their composition rating/marking quality in the context of large-scale and classroom writing assessment. To address the above research gaps, a pilot study has been conducted to explore how Chinese language teachers mark their students’ compositions in the classroom writing assessment context.

Unlike earlier TAPA studies using the paper-based approach, teacher subjects were asked to perform think-aloud marking on the tablet PCs specifically designed for the study to collect data on the nature of composition marking process. The findings of this pilot study relating to teachers’ commentary styles and patterns in an on-screen marking setting, as well as the strategies or solutions they employed to overcome the problems in the composition marking process, have been reported in other paper (Ying et al. 2014). The scope of this paper is focused on the distinguished features of such a tablet-based composition marking recording system, i.e., a camera-free think-aloud protocol instrument, followed by a brief report on the pilot study of our system.

## Use of think-aloud in the composition rating research

Think-aloud protocol analysis (TAPA) has been the most prevailing methodology employed by researchers in their studies on the rating process of both Chinese and English composition (Cumming 1990; Cumming et al. 2001, 2002; Erdosy 2004; Huot 1993; Vaughan 1991; Wolfe 1997, 2006; Wolfe and Feltovich 1994; Wolfe et al. 1998, Lumley 2006). Subject teachers were usually asked to do an on-site rating exercise on a batch of student writings, and verbally describe the thoughts going through their minds concerning the planning and decision-making procedure and all the essential considerations involved in rating concurrently. During the process, their rating performance and verbalization would be recorded, either by video-cameras or audio recorders. The recordings would then be coded and transcribed as think-aloud protocol for data analysis in the future. The merits of TAPA include the capability of recording visually imperceptible inner cognitive processes, the relatively smaller size of sample required to provide substantial qualitative indication and the alleviation of the burden of memory of the subject teachers compared to post-experiment interview.

Yet, there are a number of drawbacks of TAPA. For example, the capability of verbal representation of ones’ thinking process can vary greatly among different subjects. In order to resolve the problem, Green (1998) proposed that standardized and direct mock exercises should be provided for the subject teachers before the actual commencement of the experiment so that they can get familiar with the procedure. Besides, some scholars also challenged that in requiring the subject teachers to report their rating process, TAPA may alter their cognitive behavior. Verbalization may actually reduce their rate of deliberation since they do not usually engage in such conscious thinking while rating a composition (Lumley 2006, 2009; Crisp 2008).

In the past TAPA studies, researchers used to collect the relevant data through video-taping the composition marking process of the participant teachers and the verbalization of what went through their minds directly during the marking concurrently. In order to minimize the disturbances caused by the recording, the cameras were usually set either behind the participants or directly above their hands and the compositions. Nevertheless, there are still two intractable problems with this mode of data collection. On the one hand, the awareness of the very existence of the cameras would inevitably impose pressure on the participants and hence affect their marking performance. On the other hand, the hands and arms of the participants would keep blocking the view of the compositions from time to time, undermining the completeness of the data collected. In view of these considerations, we have developed a camera-free, tablet-based composition marking recording system that can concurrently record all the necessary data and information concerning the marking and the think-aloud process.

Accordingly, the general procedure of the pilot study is as follows. Before the commence of the composition marking experiment, the target compositions were scanned and saved as JPEG image files to the tablets in which our composition marking recording system has been installed. Then, during the experiment, each of the participants was given a tablet and requested to mark the compositions on the tablet and perform the think-aloud exercise concurrently. They were able to put down any symbols, write down any comments, and make any necessary corrections on the screen in exactly the same way as they normally did when they marked on the hardcopies.

Throughout the marking experiment, the system recorded all the symbols and words written down on the screen and, with the internal audio recorder, the verbal reports during the marking process. The researchers can then check up the recordings with the system afterward to review and pinpoint every single symbol, comment, and correction marked on the compositions and the verbal description reporting what the participants had in mind during the marking process. Furthermore, the system can also enable the researchers to measure the exact time consumed in any specific procedure in the marking process, including re-reading a particular sentence or paragraph for better understanding, thinking about certain problematic parts of the composition, jotting comments, recognizing and correcting errors and mistakes, etc.

## The tablet-based mode of TAPA

Our tablet-based Chinese composition marking system is able to record both the teacher’s written and oral feedback. In this section, the major functions of our tablet-based Chinese composition marking system are described to illustrate how this system can be used in TAPA in the research reported in this paper.

### Opening student’s composition work

**Fig. 1:**
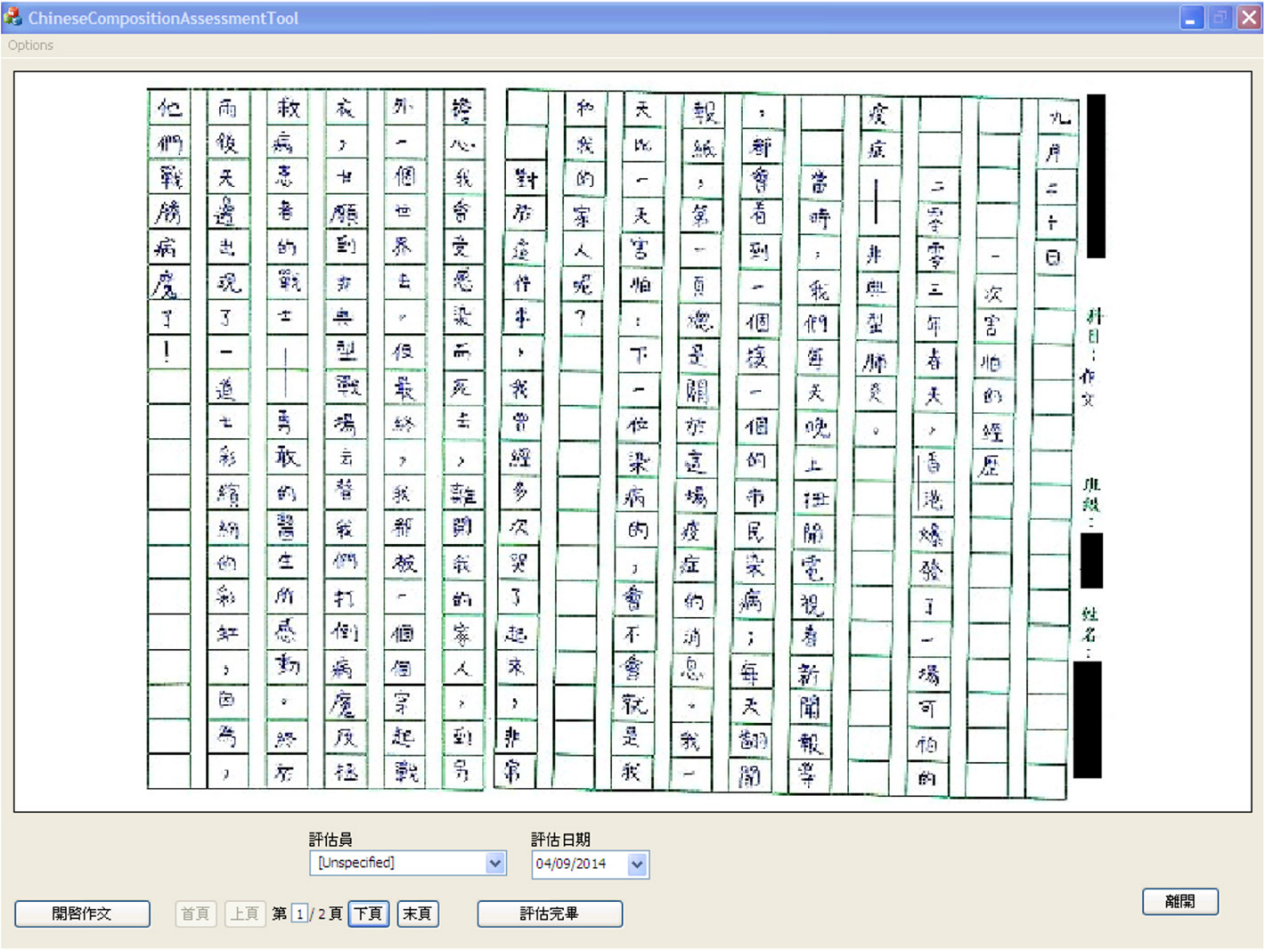
Display of student’s composition work in landscape

**Fig. 2:**
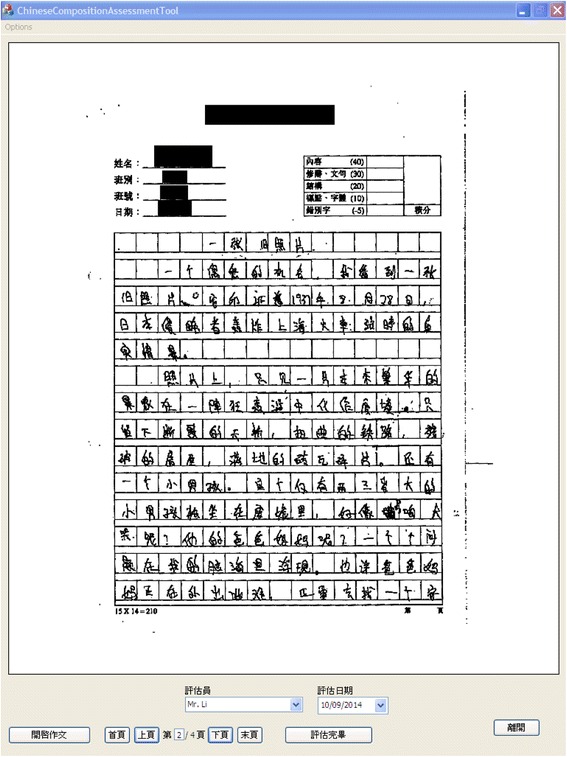
Display of student’s composition work in portrait

The Chinese composition is made by students writing on normal grid paper in the traditional way. The finished composition is scanned and stored in the commonly used JPEG format. Sometimes the composition work may consist of several pages and they will be stored with the same prefix and numbered in sequence. The teacher can run our system on a tablet and open the student’s composition. The scanned composition will be displayed using our interface. Besides, since the grid paper may be in landscape or in portrait, our system can also be run in the landscape mode or in the portrait mode in the tablet to accommodate both options, and the composition work will be displayed with the correct aspect ratio, as illustrated in Figs. 1 and 2. We also provide a list box in our interface which can show a list of possible teachers’ names, and a particular teacher can choose his/her name there to identify who performs the assessment in case the same student’s composition is marked by more than one teacher. The assessment date is also shown and will be saved together with the teacher’s feedback.

### Navigating to different pages

A student’s composition work may span several pages. As mentioned in the previous sub-section, each page will be scanned with the same prefix in the file name which is numbered in sequence. Our system will detect automatically the total number of pages exist in the student’s work after the teacher opens it with our system. In our interface, we have provided several buttons to facilitate the easy navigation of the pages. In particular, there are four buttons that allow the teacher to navigate to the previous page, the next page, the first page, and the last page.

### Providing written comments

**Fig. 3:**
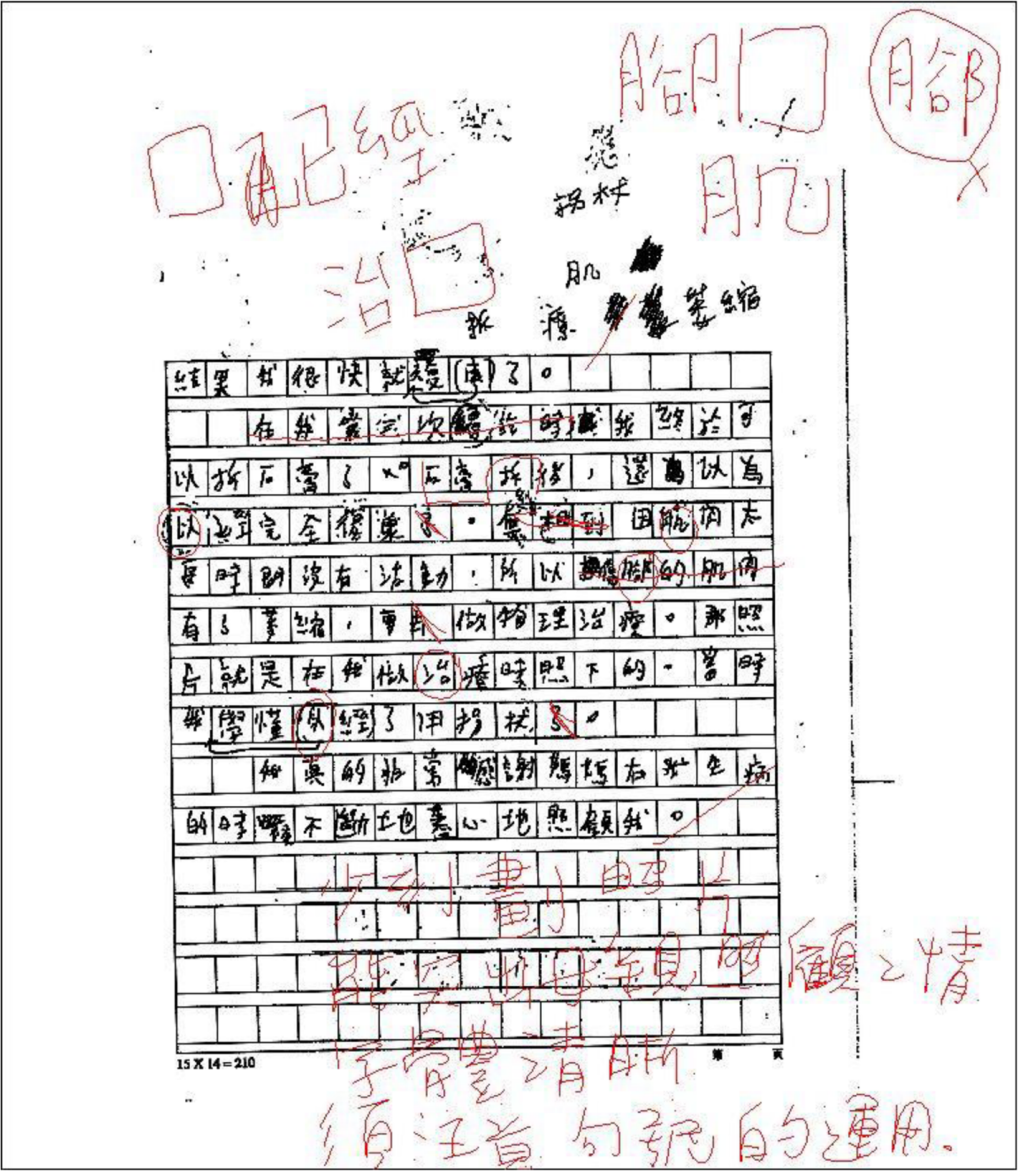
Example of student’s composition with teacher’s markup

Once the composition is displayed in our interface, the teacher can mark up on the tablet using a stylus. The teacher’s markup will be shown in red. With this function, the teacher can circle the characters that are written wrongly or underline some sentences whose structure is not well organized. The teacher can also write down explicit comments for the students to improve their work. This function simulates the actual scenario in which a teacher marks a student’s composition work on paper with a red pen such that the teacher does not need much extra time to learn to use our system for the assessment. Figure 3 illustrates an example page of student’s composition with the teacher’s markup made using our system.

### Providing oral comments

In addition to written comments, our system is able to allow the teacher to record his/her voice during the assessment such that the teacher can provide oral comments. This function can serve as a compliment to the written comments. For example, the teacher can circle a character while saying which part of the character is written wrongly. The teacher can underline some sentences and voice out how they can be revised to improve the flow. The teacher can also provide general comments to the student to provide advice about how to improve the composition skill in the future.

This voice recording feature can also be used by researchers to study the way teachers mark students’ composition. Researchers can ask the teacher to voice out what they are thinking while they are assessing the composition. Under this think-aloud approach, the teacher’s oral comments can be saved and analyzed by the researchers together with the written comments.

### Playback

The assessed composition can be played back by the researcher to analyze the teacher’s written and oral comments to study the assessment process made by the teacher. First the researcher can open the marked result using our system to view the markup by the teacher. The researcher can navigate to the previous page, next page, first page, and last page using similar buttons as introduced in the “Navigating to different pages” section. The researcher can also playback the assessment session such that the researcher can see when the teacher writes down the comments with the exact timing and listen to the teacher’s oral feedback which is synchronized with the written comments. Our interface provides control buttons to let the researcher play, pause, and stop the playback session. As mentioned previously, the researchers may ask the teachers to voice out their thoughts under the think-aloud protocol which are stored as oral comments. The researchers may analyze different strategies adopted by the teachers and try to devise novel ways for students to improve their composition skills.

## Pilot study

We have invited eight Chinese language teachers from secondary schools to test our new system of marking and participate in the read-aloud protocol for recording. The eight teachers came from four different secondary schools, six males and two females. During the testing, they needed to rate students’ Chinese compositions and simultaneously perform the read-aloud protocol for recording. Each teacher participant had to rate six student compositions on the same topic “My Experience in a Hospital”. After testing, the research team conducted a 30-min interview on each teacher participant to further understand their habits, strategies, and underlying motives in marking students’ compositions. Another major focus of the study was to gather participants’ opinions about the new system of marking with the read-aloud protocol.

Overall speaking, teacher participants responded positively to the system as it did not negatively impact their marking or cause any unnecessary interference. All participants were satisfied with their rating performance and they adopted similar rating process and method as they would normally have done in everyday marking. In conclusion, they recognized the following strengths from using our system:

1. Easy to learn and operate as little time is required to “get the hang” of it.
2. The built-in, invisible lenses alleviate the pressure on the participants as they can focus on marking and the read-aloud routine.
3. A tablet flat-screen computer takes the size of a piece of draft paper, which is no different from writing comments on students’ composition scripts in everyday marking.

Teacher participants also made useful observations and suggestions with regard to the future improvement of the system. When scanning students’ composition files, the system sometimes thickened character strokes that resemble smudged ink marks making it difficult for teacher participants to recognize the words and their meanings.

In contrast to using a red ballpoint pen to mark the composition scripts, all the teacher participants were asked to mark composition files on screen by circling out the mistakes and by writing comments with an electronic pen. As the system was new to them, some teachers found it difficult to control and it was quite slow and painstaking for them to write complete words on the screen in order to avoid broken strokes and incomplete characters. As the electronic pen is black in color which is less conspicuous than a red ballpoint pen, teacher participants had to spend more time checking out the word number, going through previous corrections, etc. to substantiate their remarks and overall comments.

Some teachers had the distinct habit of reading through the whole composition once to form a general impression before marking it. Others had to go through previous marked compositions or scoring records to make future marking and scoring decisions. The current electronic design was found to be restrictive in supporting these functions. In other words, while rating student D’s composition, the system did not make allowance for the teacher to simultaneously retrieve students A, B, and C’s compositions to draw comparisons in order to reach decisions about student D’s score. Under the present system configuration, if the teacher departed from the current file, the whole process of image and sound recording would automatically be terminated. In fact, the teacher had to save the current file before opening other target files. Alternatively, s/he could revert to the marking process recorded earlier to access the JPEG files of compositions marked previously. After checking out the previous files, s/he could then reopen student D’s text file (unmarked composition) to rate it and then save it as a new file.

Simply put, simultaneous “multiple marking” or “multiple reading” of a few compositions is not possible with the current system. In this respect, the researcher still needs to rely on an external recording pen or participants’ self reporting or description of the marking process. This could be identified as the major drawback of the present system.

This system was first designed for the pilot scheme of the current research study. As it is still in its initial stage of development, there are many shortcomings which require further enhancement and fine-tuning. After collecting participants’ opinions and suggestions, the research team would conduct focus group interviews, questionnaire surveys, and other relevant tests to further establish the validity and reliability of using the think-aloud protocol as an effective research and test tool.

## Conclusions and future work

In a nutshell, our camera-free composition marking recording system can (1) effectively eliminate the pressure of the participants facing the cameras; (2) allow the researchers to record the whole marking and think-aloud process in a highly accurate, detailed, and systematic manner, and hence (3) provide a convenient and powerful database for future study.

One of the co-authors of this paper and her research team asked Chinese language teachers to use think-aloud protocol to record their process of marking students’ composition (Ying et al. 2014). In their studies, participant teachers were positive about the tablet-based composition marking platform as it did not cause major inconvenience to them in the marking process. They reported that the influence only existed in marking speed level, and the system had no influence to the procedure of their marking process. This coincides with Crisp’s findings (Crisp 2008). Crisp arranged for six markers to use think-aloud protocol to record their correction of compositions in a writing examination. Results revealed that their marking speed was slightly slower than usual, and their feedback and correction were harsher than usual.

Since the present study is only one of the few attempts to explore the nature of the composition marking process of Chinese writing teachers with the method of TAPA conducted in a computer-based assessment environment, more empirical studies should be done on this topic so as to enhance the reliability and validity and, most importantly, applicability of the findings.
